# Supplementary material for: ULK3 regulates cytokinetic abscission by phosphorylating ESCRT-III proteins
Source: eLife. 2015 May 26;4:e06547. doi: 10.7554/eLife.06547 (PMC4475061; doi:10.7554/eLife.06547)
Supplement: Figure 1—source data 1. — DOI: http://dx.doi.org/10.7554/eLife.06547.004 [file elife06547s001.docx]

**Figure 1-source data 1: Data Collection and Refinement Statistics for the ULK3 MIT2:IST1 MIM1 Complex.**

|  | **Native** | **SeMet (SAD)** |
| --- | --- | --- |
| **Data collection** |  |  |
| Space group | R32:H | P3_1_2_1_ |
| Cell constants:  *a,b, c* (Å), α, β, γ (degrees) | 79.12, 79.12, 96.62, 90, 90,120 | 82.68, 82.68, 90.15, 90, 90,120 |
|  |  |  |
| Wavelength (Å) | 1.000 | 0.97905 |
| Resolution (Å) | 40.50-1.38 (1.43-1.38) | 40.0-2.10 (2.17-2.10) |
| Total observations | 670968 (6837) | 266771 (11948) |
| Unique observations | 23,612 (1025) | 20,820 (1141) |
| *R*_merge_^b^ | 0.066 (1.124) | 0.146 (2.612) |
| *R_meas_^c^* | 0.067(1.302) |  |
| *R_pim_^d^* | 0.013 (0.638) | 0.061 (1.345) |
| *CC_1/2_^e^* | 1.000(0.842) |  |
| *I* / σ(*I)* | 66 (1.5) | 12 (0.7) |
| Completeness (%) | 99.1 (85.2) | 98.7 (81.9) |
| Redundancy | 28.4 (6.7) | 12.8 (8.3) |
| Mosaicity | 0.18 | 0.12 |
|  |  |  |
| **Refinement** |  |  |
| Resolution (Å) | 20.0-1.38 (1.43-1.38) |  |
| No. unique reflections | 23,588 (2155) |  |
| R-free test reflections | 1993 (8.49%) |  |
| Wilson B-factor (Å) | 22.5 |  |
| Anisotropy | 0.622 |  |
| F_0_,F_c_ correlation | 0.97 |  |
| *R*_cryst_^f^/ *R*_free_ ^g^ | 0.151/0.180 (0.325/0.376) |  |
| No. atoms | 900 |  |
| Protein | 103 residues |  |
| Solvent | 67 |  |
| Ligands (sulfate, cobalt) | 6 |  |
| Average *B*-factor (Å^2^) | 39.0 |  |
| r.m.s. deviations |  |  |
| Bond lengths (Å) | 0.009 |  |
| Bond angles (°) | 1.128 |  |
| Ramachandran |  |  |
| Favored (%) | 100 |  |
| Outliers (%) | 0.0 |  |
| Rotamer outliers | 0.0 |  |
| Clashscore | 0.60 |  |
| Molprobity score | 0.7 |  |

Native and SeMet data were each collected from single crystals.

Values in parentheses correspond to the high-resolution shell.

Refinement statistics were determined by PHENIX-dev-1760 and MolProbity4.1.

^a^ Data were collected at the SSRL.

^b^ R_merge_ = (∑|(I − ‹I›)|)/(∑I), where ‹I› is the average intensity of multiple measurements[^1^](#_ENREF_1)^,^[^2^](#_ENREF_2).

^c^ R_meas_ = (∑(N/N-1)^1/2^(I − ‹I›)|)/(∑I), where ‹I› is the average intensity of multiple measurements.

^d^ R_pim_ = (∑(1/N-1)^1/2^(I − ‹I›)|)/(∑I), where ‹I› is the average intensity of multiple measurements[^3^](#_ENREF_3).

^e^ CC_1/2_ = ∑(x -‹x›) (y -‹y›)/[∑(x -‹x›)^2^ ∑(y -‹y›)^2^ ]^1/2^, where ‹I› is the average intensity of multiple measurements^[4](#_ENREF_4)^.

^f^ R_cryst_ = (∑|F_obs_ − F_calc_|)/(∑|F_obs_|).

^g^ R_free_ is the R_cryst_ based on >1000 of the reflections that were excluded from refinement.

1. Diederichs, K. & Karplus, P.A. Improved R-factors for diffraction data analysis in macromolecular crystallography. *Nature structural biology* 4, 269-75 (1997).

2. Weiss, M.S., Hilgenfeld, R. On the use of the merging R factor as a quality indicator for X-ray data. *Journal of Applied Crystallography* 30, 203-205 (1997).

3. Weiss, M.S. Global indicators of X-ray data quality. *Journal of Applied Crystallography* 34, 130 (2001).

4. Karplus, P.A. & Diederichs, K. Linking crystallographic model and data quality. *Science* 336, 1030-3 (2012).
