# Supplementary material for: ULK3 regulates cytokinetic abscission by phosphorylating ESCRT-III proteins
Source: eLife. 2015 May 26;4:e06547. doi: 10.7554/eLife.06547 (PMC4475061; doi:10.7554/eLife.06547)
Supplement: Supplementary file 1. — Supplementary information. Includes list of plasmids used in this study, siRNA sequences, primary antibodies, stable cell lines used in this study. DOI: http://dx.doi.org/10.7554/eLife.06547.046 [file elife06547s002.docx]

**Supplementary Information**

**Lists of plasmids used in this study**

Vectors encoding yeast two-hybrid, pCR3.1/YFP and pCAGGS/GST-tagged ESCRT-III and ESCRT-III-associated proteins have been described previously ([Agromayor et al., 2009](#_ENREF_1); [Martin-Serrano et al., 2003](#_ENREF_5)).

**a. Yeast 2-Hybrid vectors**

| Construct | Insert | Source | Backbone | Tags |
| --- | --- | --- | --- | --- |
| HB18-ULK3 | ULK3 full length wild type | IMAGE clone 8862706 | pHB18 (VP16 activation domain) | HA |
| HB18-ULK3 K44H | ULK3 full length catalytically inactive | IMAGE clone 8862706 | pHB18 (VP16 activation domain) | HA |
| HB18-ULK3 M434D | ULK3 full length MIT2 MIM1-binding mutant | IMAGE clone 8862706 | pHB18 (VP16 activation domain) | HA |
| HB18-ULK3 V338D | ULK3 full length MIT1 MIM1-binding mutant | IMAGE clone 8862706 | pHB18 (VP16 activation domain) | HA |
| HB18-IST1 | IST1 full length wild type | Accession number BC103745 | pHB18 (VP16 activation domain) | HA |
| HB18-IST1 4SA | IST1 full length, phospho-deficient mutant: S4A, S99A, S153A and S214A | Accession number BC103745 | pHB18 (VP16 activation domain) | HA |

**b. Mammalian Expression Vectors**

| Construct | Insert | Source | Backbone | Tags | Internal ID | |
| --- | --- | --- | --- | --- | --- | --- |
| CHMP1A-myc | CHMP1A full length | Accession  NP_002759 | pCAG-Cmyc | C-myc | WISP08-77 |  |
| CHMP1B-myc | CHMP1B full length | Accession AAG01449 | pCAG-Cmyc | C-myc | WISP08-78 |  |
| CHMP2A-myc | CHMP2A full length | Accession NP_002759 | pCAG-Cmyc | C-myc | WISP08-97 |  |
| CHMP2B-myc | CHMP2B full length | Accession AF151842 | pCAG-Cmyc | C-myc | WISP14-122 |  |
| CHMP3-myc | CHMP3 full length | Accession AF219226 | pCAG-Cmyc | C-myc | WISP08-99 |  |
| CHMP4A-myc | CHMP4A full length | Accession NM_014169.2 | pCAG-Cmyc | C-myc | WISP08-79 |  |
| CHMP4B-myc | CHMP4B full length | Accession NM_176812.4 | pCAG-Cmyc | C-myc | WISP08-80 |  |
| CHMP4C-myc | CHMP4C full length | Accession NM_152284.3 | pCAG-Cmyc | C-myc | WISP08-81 |  |
| CHMP5-myc | CHMP5 full length | Accession NM_016410.5 | pCAG-Cmyc | C-myc | WISP11-321 |  |
| CHMP6-myc | CHMP6 full length | Accession NM_024591.4 | pCAG-Cmyc | C-myc | WISP08-83 |  |
| CHMP7-myc | CHMP7 full length | Accession NM_152272.3 | pCAG-Cmyc | N-myc | WISP08-94 |  |
| Myc-IST1 | IST1 full length | Accession BC103745 | pCAG-Nmyc | N-OSF | WISP07-77 |  |
| OSF-ULK3 | ULK3 full length wild type | IMAGE clone 8862706 [1] | pCAG-N-OSF | N-OSF | WISP14-109 |  |
| OSF-ULK3 K44H | ULK3 full length catalytically inactive | IMAGE clone 8862706 [1] | pCAG-N-OSF | N-OSF | WISP14-114 |  |
| OSF-ULK3 K139R | ULK3 full length catalytically inactive | IMAGE clone 8862706 [1] | pCAG-N-OSF | N-OSF | WISP14-113 |  |
| OSF-ULK3 1-270 | ULK3 kinase domain | IMAGE clone 8862706 [1] | pCAG-N-OSF | N-OSF | WISP14-110 |  |
| OSF-ULK3 277-472 | ULK3 C-terminal domain | IMAGE clone 8862706 [1] | pCAG-N-OSF | N-OSF | WISP14-111 |  |
| OSF-ULK3 277-449 | ULK3 tandem MIT domains | IMAGE clone 8862706 [1] | pCAG-N-OSF | N-OSF | WISP14-112 |  |
| GST-ULK3 | ULK3 full length wild type | IMAGE clone 8862706 | pCAGGS-GST | N-GST |  |  |
| GST-ULK3 K44H | ULK3 full length catalytically inactive | IMAGE clone 8862706 | pCAGGS-GST | N-GST |  |  |
| GST-ULK3 V338D | ULK3 full length MIT1 MIM1-binding mutant | IMAGE clone 8862706 | pCAGGS-GST | N-GST |  |  |
| GST-ULK3 M434D | ULK3 full length, MIT2 MIM1-binding mutant | IMAGE clone 8862706 | pCAGGS-GST | N-GST |  |  |
| YFP-IST1 | IST1 full length | Accession number BC103745 | pCR3.1/YFP | N-YFP |  |  |
| pNG72-ULK3^R^ | ULK3 full length siRNA-resistant [2] wild type | IMAGE clone 8862706 | pNG72 retroviral packaging vector | - |  |  |
| pNG72-ULK3^R^ K44H | ULK3 full length siRNA-resistant [2], catalytically inactive | IMAGE clone 8862706 | pNG72 retroviral packaging vector | - |  |  |
| pNG72-ULK3^R^ V338D | ULK3 full length siRNA-resistant [2], MIT1 MIM1-binding mutant | IMAGE clone 8862706 | pNG72 retroviral packaging vector | - |  |  |
| pNG72-ULK3^R^ M434D | ULK3 full length siRNA-resistant [2], MIT2 MIM1-binding mutant | IMAGE clone 8862706 | pNG72 retroviral vector | - |  |  |
| pCMS28 OSHA-ULK3 | ULK3 full length wild type | IMAGE clone 8862706 | pCMS28 retroviral vector | N-HA |  |  |
| pCMS28 OSHA-ULK3 K44H | ULK3 full length catalytically inactive | IMAGE clone 8862706 | pCMS28 retroviral vector | N-HA |  |  |
| pCMS28 OSHA-ULK3 M434D | ULK3 full length MIT2 MIM1-binding mutant | IMAGE clone 8862706 | pCMS28 retroviral vector | N-HA |  |  |
| pCMS28-IST1^R^ | IST full length siRNA resistant [3] | Accession number BC103745 | pCMS28 retroviral vector | - |  |  |
| pCMS28-IST1^R^ 4SE | IST1 full length phosphomimetic siRNA resistant [3]: S4E, S99E, S153E and S214E | Accession number BC103745 | pCMS28 retroviral vector | - |  |  |
| pCMS28-IST1^R^ 4SA | IST1 full length phospho-deficient siRNA resistant [3] | Accession number BC103745 | pCMS28 retroviral vector | - |  |  |
| pCMS28 BirA-ULK3 | ULK3 full length wild type | IMAGE clone 8862706 | pCMS28 retroviral vector | N-BirA |  |  |
| LentiCRISPRv2-ULK3-1 | gRNA targeting ULK3 (set 1) | Sequences given in main text | LentiCRISPRv2 packaging vector | - |  |  |
| LentiCRISPRv2-ULK3-4 | gRNA targeting ULK3 (set 2) | Sequences given in main text | LentiCRISPRv2 packaging vector | - |  |  |

**c. Bacterial Expression Vectors**

| Construct | Insert | Source | Backbone | Tags | Internal ID |
| --- | --- | --- | --- | --- | --- |
| GST-ULK3(MIT)_2_ | ULK3 tandem MIT domains; residues 277-449, wild-type | IMAGE clone 8862706 [1] | pGEX-PP | N-GST, Precission Protease- cleavable | WISP14-117 |
| GST-ULK3(MIT)_2_ V338D | ULK3 tandem MIT domains; residues 277-449, MIT1 MIM1-binding mutant | IMAGE clone 8862706 [1] | pGEX-PP | N-GST, Precission Protease- cleavable | WISP14-119 |
| GST-ULK3(MIT)_2_ M434D | ULK3 tandem MIT domains; residues 277-449, MIT2 MIM1-binding mutant | IMAGE clone 8862706 [1] | pGEX-PP | N- GST, Precission Protease- cleavable | WISP14-118 |
| GST-ULK3 MIT2 | ULK3 residues 359-449, wild-type | IMAGE clone 8862706 [1] | pGEX-PP | N-term GST, Precission Protease cleavable | WISP14-120 |
| pGEX-TEV-IST1_303-366_ | IST1 MIMs, 303-366, wild-type | BC103745 | pGEX-TEV | N-term GST, TEV- cleavable | WISP07-210 |
| HIS-SUMO-IST1_316-366_ | IST1 MIMs, 316-366, wild-type non-native N-term ‘GC’ for labeling | BC103745 | pCA528 | N-term SUMO, UBPL1- cleavable; | WISP14-121 |

[1] Site-directed mutagenesis was used to insert V444 and K445 into IMAGE clone 8862706 (isoform 3) so that the expressed protein sequence matches mRNA seq NM_001099436.1 (isoform 1).

[2] ULK3 siRNA-resistant (ULK3^R^) version was generated by introduction of the silent mutations C308C, K309K, A310A, L311L, D312D and F313F within the ULK3 siRNA targeting sequence.

[3] IST1 siRNA-resistant (IST1^R^) was generated by insertion of the silent mutations L166L, I167I, E168E, I169I, A170A, K171K and N172N within the IST1 siRNA targeting sequence.

**siRNA sequences used in this study, related to Experimental Procedures.**

| **Protein** | **Sense sequence** | **Source** |
| --- | --- | --- |
| Non-targeting | ON-TARGET plus siControl non-targeting pool | Dharmacon (D-001810-10-20) |
| CHMP4C | CTCACTCAGATTGATGGCACA | Qiagen (Cat. number SI04279674) |
| ULK3 | GCAAGGCUCUGGACUUCUU | Dharmacon (Cat. number D-004949-01) |
| ULK3-4 | CACGGAGATTGAGATCCTCAA | Qiagen (Cat. Number 1027415) |
| TSG101 | CCUCCAGUCUUCUCUCGUC | Dharmacon (Custom order siRNA) |
| NUP153 | GGACUUGUUAGAUCUAGUU | Dharmacon (Custom order siRNA) |
| IST1 | TCGCCTTAAACTATTGGAGAA | Qiagen (Cat No. SI0045241) |

**Primary antibodies used in this study, related to Experimental Procedures.**

| Antibody | Source | Conditions |
| --- | --- | --- |
| Mouse α-Tubulin (clone DM1a) | Sigma | WB: 1:5,000 in 1% milk; nitrocellulose |
| Mouse α-HIV-1 p24  (clone 183-H12-5C) | National AIDS Research and Reference Reagent Program | WB: 1:100 in 1% milk; nitrocellulose |
| Mouse α-TSG101 (clone 4A10) | Abcam | WB: 1:1,000 in 1% milk; nitrocellulose |
| Mouse α-HA.11 | Covance | IF: 1:1,000 in 1% BSA in 1X PBS  WB: 1:1,000 in 1% milk; nitrocellulose |
| Rabbit α-HA | Rockland Immunochemicals | WB: 1:500 in 1% milk; nitrocellulose |
| Rabbit α-Phospho-Histone H3 (Ser10) | Cell Signalling Technology | WB: 1:500 in 1% milk; nitrocellulose |
| Rabbit α-Tubulin | Cell Signalling Technology | IF: 1:100 in 1% BSA in 1X PBS |
| Rabbit α-IST1 | (University of Utah; Bajorek et al., 2009) | IF: 1:10,000 in 1% BSA in 1X PBS |
| Rabbit α-ULK3 | Epitomics, Abcam (EPR4888) | WB: 1:1,000 in 1% milk; nitrocellulose |
| Mouse α-CHMP4C | EMBL Monoclonal Antibody Core Facility (Carlton et al., 2012) | WB: 1:200 in 1% milk; nitrocellulose |
| Mouse α-GST | EMBL Monoclonal Antibody Core Facility | WB: 1:1000 in 1% milk; nitrocellulose |
| Mouse α-ULK3 | Mouse hybridoma cells were produced by the EMBL Monoclonal Antibody Core Facility using bacterially-expressed GST-ULK3 fusion lacking both MIT domains (residues 1-267) and a monoclonal antibody detecting endogenous ULK3 protein was selected | IF: 1:400 in 1% BSA in 1X PBS  WB: 1:1,000 in 1% milk; nitrocellulose |
| Mouse α-NUP (414) | Abcam | WB: 1:1,000 in 1% milk; nitrocellulose |
| Rabbit α-HSP90 (H114) | Santa Cruz Biotechnology | WB: 1:5,000 in 1% milk; nitrocellulose |
| Rabbit α-ULK3 (S12) | Santa Cruz Biotechnology | IF: 1:150 in 3% FCS in 1X PBS |
| Mouse α-AIM-1 (Aurora B) | BD Biosciences | WB: 1:500 in 1% milk |
| Rabbit α-Aurora B pT232 | Rockland Immunochemicals | IF: 1:500 in 1% BSA in 1X PBS |
| Rabbit α-IST1 | Proteintech | WB: 1:1,000 in 1% milk; nitrocellulose |
| Rabbit α-MKLP1 | Santa Cruz Biotechnology | IF: 1:450 in 1% BSA in 1X PBS |
| Mouse α-GFP (7.1/13.1) | Roche | IF: 1,1000 in 1% BSA in 1X PBS  WB: 1:5,000 in 1% milk; nitrocellulose |
| Mouse α-human HLA ABC:FITC (MCA81F) | AbD Serotec | FACS: 1:100 in 2% FBS in 1X PBS |
| Mouse α-Flag (clone M2) | Sigma | WB: 1:10,000 in 5% milk; PVDF |
| Mouse α-Myc (clone 4A6) | EMD Millipore | WB: 1:1,000 in 5% milk; PVDF |

**Stable cell lines generated in this study, related to Experimental Procedures.**

| **Cell Line** | **Promoter driving transgene** |
| --- | --- |
| HeLa mCh-Tubulin/pNG72-ULK3^R^ WT and K44H/V338D/M434D mutants | MSCV LTR and CMV IRES promoters |
| HeLa pCMS28-IST1^R^ WT and 4SA/4SE mutants | MSCV LTR and CMV IRES promoters |
| HeLa pCMS8-OSHA ULK3 WT and K44H/M434D mutants | MSCV LTR and CMV IRES promoters |
| HeLa pNG72-YFP-Tubulin | MSCV LTR and CMV IRES promoters |
| LentiCRISPRv2-ULK3 gRNA-1 | U6 promoter |
| LentiCRISPRv2-ULK3 gRNA-4 | U6 promoter |
| pCMS28 BirA-ULK3 | MSCV LTR and CMV IRES promoters |

**Previously described cell lines used in this study, related to Experimental Procedures.**

| **Cell Line** | **Reference** |
| --- | --- |
| HeLa mCherry-Tubulin | ([Agromayor et al., 2009](#_ENREF_1)) |
| HeLa mCherry-Tubulin/HA-CHMP4C | ([Carlton et al., 2012](#_ENREF_2)) |
| HeLa mCherry-Tubulin/HA-CHMP4CδINS | ([Carlton et al., 2012](#_ENREF_2)) |
| HeLa mCherry-Tubulin/ YFP-LAP2β | ([Carlton et al., 2012](#_ENREF_2)) |
| HeLa GFP-CHMP4B | Kind gift from Prof Paul Bieniasz (The Rockefeller University, USA)([Jouvenet et al., 2011](#_ENREF_4)) |
| HeLa GFP-CHMP4C | Kind gift from Prof Paul Bieniasz (The Rockefeller University, USA) ([Jouvenet et al., 2011](#_ENREF_4)) |
| HeLa KSHV K3 | Kind gift from Paul Lehner (University of Cambridge) ([Hewitt et al., 2002](#_ENREF_3)) |
| HT1080/Tetherin (THN)-HA | ([Pardieu et al., 2010](#_ENREF_6)) |
| HT1080/THN-HA KSHV K5 | ([Pardieu et al., 2010](#_ENREF_6)) |
